# Intravenous Immunoglobulin Treatment in Kawasaki Disease Decreases the Incidence of Myopia

**DOI:** 10.3390/jcm10071381

**Published:** 2021-03-30

**Authors:** Hun-Ju Yu, Meng-Ni Chuang, Chiao-Lun Chu, Pei-Lin Wu, Shu-Chen Ho, Ho-Chang Kuo

**Affiliations:** 1 Department of Ophthalmology, Kaohsiung Chang Gung Memorial Hospital and Chang Gung University College of Medicine, Kaohsiung 83301, Taiwan; angelayu@cgmh.org.tw (H.-J.Y.); choiyamapi@livemail.tw (M.-N.C.); 2 Department of Public Health, College of Health Sciences, Kaohsiung Medical University, Kaohsiung 83301, Taiwan; o911079251@gmail.com (C.-L.C.); annajudy1@gmail.com (P.-L.W.); 3 Kawasaki Disease Center and Department of Pediatrics, Kaohsiung Chang Gung Memorial Hospital and Chang Gung University College of Medicine, Kaohsiung 83301, Taiwan; cho0702@gmail.com

**Keywords:** myopia, Kawasaki disease, intravenous immunoglobulin

## Abstract

Kawasaki disease (KD) is a systemic vasculitis that primarily affects children under the age of 5 years old. The most significant complication is coronary artery lesions, but several ocular manifestations have also been reported. Recently, one study revealed an increasing incidence of myopia among KD patients. Therefore, the aim of this study was to assess the difference in myopic incidence between Kawasaki disease (KD) patients treated with aspirin and intravenous immunoglobulin (IVIG). *Materials and methods:* We carried out a nationwide retrospective cohort study by analyzing the data of KD patients (ICD-9-CM code 4461) from Taiwan’s National Health Insurance Research Database (NHIRD) during the period of 1996–2013. *Results:* A total of 14,102 diagnosed KD were found in Taiwan during the study period. After excluded missing data, treatment strategy and age distribution, a total of 1446 KD patients were enrolled for analysis including 53 of which received aspirin (without IVIG) and 1393 of which were treated with IVIG. Patients who had myopia, astigmatism, glaucoma, cataract, etc. prior to their KD diagnosis were excluded. The age range was 0 to 6 years old. According to the cumulative curves, our results demonstrated that the myopic incidence in the IVIG group was significantly lower than the aspirin group (hazard ratio: 0.59, 95% confidence intervals: 0.36~0.96, *p* = 0.02). Treatment with IVIG for KD patients may have benefit for myopia control. *Conclusion:* Compared to aspirin, IVIG may decrease the myopic risk in KD patients. However, it needs further investigation including clinical vision survey of myopia due to the limitations of this population-based study.

## 1. Introduction

With the myopic population surging around the world [1], myopia has begun to receive more attention. The prevalence of myopia is high in East Asia, and the number reported in Taiwan is particularly remarkable, with around 10% of 6-year-old children having myopia [2]. Since refractive error progresses rapidly in childhood, high myopia, which is now defined as −5.0 diopters (D), is common. Complications of myopia, such a macular degeneration secondary to pathologic myopia, is a threat to life-long vision [3]. The key mechanism of myopic progression is elongation of the eyeball. Some researchers proposed a relationship with scleral tissue loss and scleral remodeling [4], while others are trying to determine how inflammation can weaken the sclera [5].

In 1974, Kawasaki et al. was the first to report an acute febrile mucocutaneous lymph node syndrome [6], which was later named Kawasaki disease (KD). The diagnostic criteria of KD consisted of persistent fever and four out of five clinical features, including, conjunctivitis, oral mucosa change, necklymphadenopathy, limbs indurations and skin rashes [7,8]. Coronary artery lesion (CAL) is the most devastating sequela of KD [8]. The mortality rate after the acute phase was higher among those with cardiac complication compared to those without [9]. The incidence of KD in Asia, including Taiwan and Japan, is around 70 to 248 per 100,000 children [10,11,12], while that in the United States is 20 per 100,000 [13]. With a relatively high population of KD, proper treatment for these children is crucial. Mainstream management consists of adopting aspirin and intravenous immunoglobulin (IVIG) to control inflammation. However, the application of these treatments is still debated [14].

The most common ocular manifestation of KD is conjunctivitis, one of its diagnostic criteria. Other ocular findings that have been previously reported include anterior uveitis, papilledema, conjunctival hemorrhage, etc. [15,16]. An increased incidence of myopia has recently been discovered in KD cases [17]. A possible mechanism is the inflammatory character of KD. The systemic vascular complications and response to IVIG for KD are related to the extent of inflammations and that this extent of inflammatory cytokines might be reflected on the eye. [18] If so, the anti-inflammatory treatment of KD may affect the development of myopia. The purpose of this study was to determine the difference of myopic incidence between KD patients treated with aspirin alone and those treated with IVIG.

## 2. Materials and Methods

### 2.1. Subjects

Our subjects were obtained from the Taiwan’s National Health Insurance Research Database (NHIRD) based on the International Classification of Diseases, Ninth Revision, Clinical Modification (ICD-9-CM), as in our previous reports [19]. We further retrieved information from their medical records, including demographic data, diagnosis, and treatment. Children aged from 0 to 6 years old (born during the period from 2000 to 2005) were included if diagnosed with KD (ICD-9-CM: 446.1) at an out-patient department or at admission during the period of year1996 to 2013. We excluded any cases that were missing information of a personal character, such as gender and age. Furthermore, those who were diagnosed with myopia (ICD-9-CM: 367.1), chorioretinal inflammation, scars, and other disorders of the choroid (ICD-9-CM: 363), disorders of the iris and ciliary body (ICD-9-CM: 364), glaucoma (ICD-9-CM: 365), cataract (ICD-9-CM: 366), astigmatism (ICD-9-CM: 367.2), anisometropia and aniseikonia (ICD-9-CM: 367.3), ptosis (ICD-9-CM: 374.3), and/or congenital nystagmus (ICD-9-CM: 379.51) prior to KD diagnosis were removed from this study. Based on the management they received, these patients were divided into two groups, one that received aspirin (without IVIG), while the other received IVIG (with or without aspirin). Myopia (ICD-9-CM: 367.1) was set as the outcome.

### 2.2. Statistical Analysis

To compare the characteristics of the aspirin alone and IVIG groups, including follow-up years, gender, and age, the t-test was applied. Cox regression analysis was used to calculate the hazard ratio (HR) and 95% confidence intervals (CIs) of myopia for both groups. Regarding the cumulative incidence of myopia, we used the Log-rank test to depict the results of myopia development in the aspirin and IVIG groups. We applied SAS 9.4 statistical software (SAS Institute Inc., Cary, NC, USA) for all the statistical analyses in this study. In the two-tailed test, a value of *p* < 0.05 was considered statistically significant.

## 3. Results

**Figure 1:**
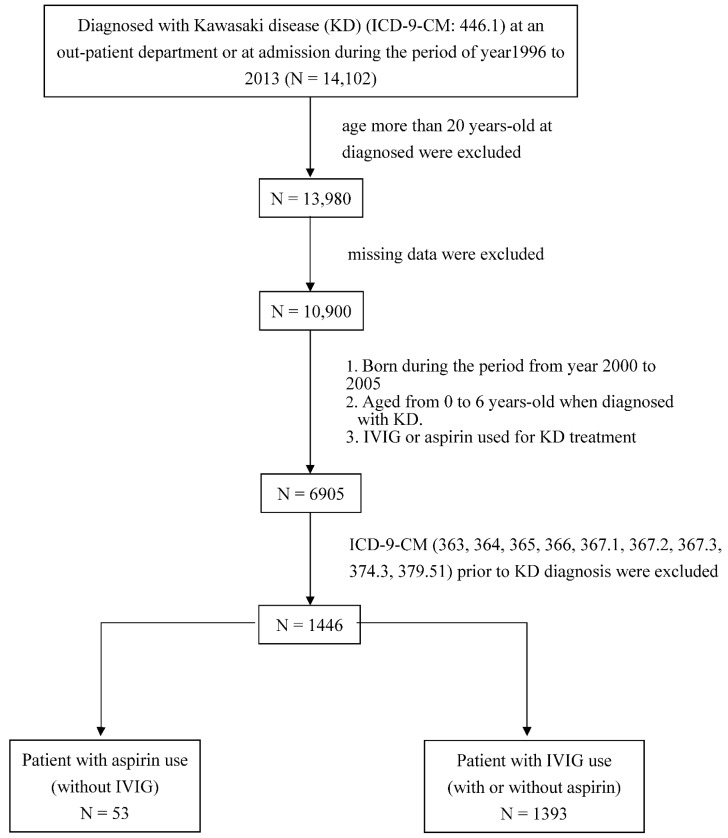
Flowchart of study subjects collected.

A total of newly diagnosed with KD were 14,102 during the study period of year 1996–2013. The flowchart of this study was showed in Figure 1. This study included a total of 1446 subjects for analysis. Of those, 53 patients were treated with aspirin (without IVIG), while 1393 patients received IVIG (with or without aspirin). The characteristics of these cases are summarized in Table 1. The mean follow-up time for the aspirin group was 3.67 ± 1.34 years, and that of the IVIG group was 4.00 ± 1.41 years. The distribution of gender and age between these two groups showed no significant difference (*p* > 0.05). Male gender showed significant higher in IVIG group than aspirin group (male/female: 50.94/49.06 vs. 64.32/36.68%, *p* = 0.046).

**Figure 2:**
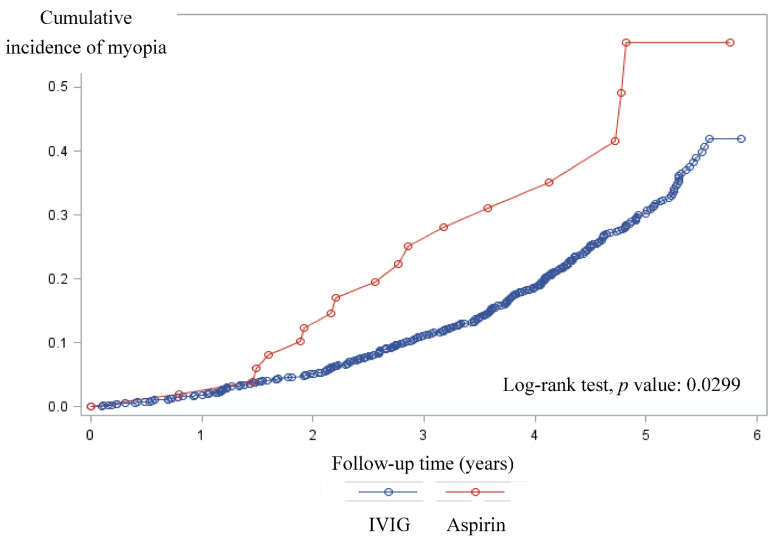
Cumulative incidence curves of myopia for Kawasaki disease groups.

The cumulative curves of myopic incidence in the aspirin group and IVIG group are shown in Figure 2. According to the Log-rank test, the cumulative incidence of myopia in the aspirin group was higher than the IVIG group (*p* = 0.02).

A comparison of the crude adjusted hazard ratio (HR) of myopia between the two groups indicated that the incidence of myopia in the aspirin group was significantly higher (Table 2) than in the IVIG group. Male gender (boys) had higher risk of myopia than female gender (girls) in both aspirin group (*p* < 0.0001) and IVIG group (*p* < 0.01). The myopic incidence increased with age: children 1–3 years old reported a 1.89-fold risk compared to the 1-year-old group (*p* < 0.0001). Those aged from 4 to 6 years old had an incidence 10.1 times greater than the 1-year-old group (*p* < 0.0001).

## 4. Discussion

King et al. discovered the correlation between myopia and Kawasaki disease in 2017 [17]. The KD group had an overall incidence of myopia around 4.3 per 100 person-years, while the non-KD group had an incidence of 3.3 per 100 person-years. They recruited their subjects from the NHIRD before 2010, while the prevalence of myopia in pre-school children was around 6–7% in Taiwan. Comparing children of the same age in 2017, the year with the latest report from Taiwan’s Ministry of Health and Welfare, the prevalence increased to 7–9%. In other words, the KD population would have an expanded proportion of myopia among it, along with the growing myopia population.

Inflammation is one of the proposed mechanisms of myopic development. Several diseases involving inflammation, such as diabetes mellitus rheumatoid disease, have been reported to have a positive correlation with myopia or to have increased the risk of myopia. An observational study performed by Fledelius et al. in 1983 reported that myopia is more common in diabetics than in non-diabetics (37.9% vs. 27.5%) [20]. Upcoming studies also support that diabetes mellitus is a risk factor for myopia [21]. According to the Los Angeles Latino Eye Study, compared with those without juvenile chronic arthritis (JCA), the refractive error is more negative in the JCA group [21]. Through epidemiologic observation, Lin el al. discovered that type 1 diabetes mellitus, uveitis, and systemic lupus erythematosus (SLE) tend to have a higher incidence of myopia [5]. Form-deprivation animal models were used to test and verify this concept. The link between myopia and inflammation was indicated since the up regulation of the inflammatory response can accelerate myopic development, while suppressing inflammation can otherwise inhibit myopic progression. Another study conducted by Wei et al. found that children with allergic conjunctivitis were at a higher risk of having myopia [22]. Animal model experiments have indicated that inflammation of the ocular surface, such as allergies with mast cell degranulation, may lead to sequential retinal inflammation, which may promote myopic progression based on a previous study. The idea that myopia and inflammation are connected was further supported in another way. Multiple evanescent white dot syndrome and multifocal choroiditis are also associated with myopia. The pathophysiology of these disease is choriocapillaropathy, which is secondary to inflammation and leads to the irregular perfusion of retina [23]. With chronic inflammation, inflammatory choroidal neovascularization (CNV), a sight-threatening lesion, is likely to occur [24].

Aspirin, a non-steroidal anti-inflammatory drug, had been used to treat KD patients for decades, even before IVIG. Aspirin has a different role according to its dosage; a high dose (greater than 30 mg/kg/day) has an anti-inflammation effect, while a low dosage (less than 5 mg/kg/day) has an anti-platelet effect. The aspirin group of KD patients in this study that did not receive IVIG may have been due to the fever subsiding while making diagnosis. Meanwhile, IVIG, another first-line standard therapy for KD, is adopted to prevent sequential complications by controlling inflammation [25]. The vascular complications and response to IVIG for KD are related to the extent of inflammation and that this extent of inflammation might be reflected on the eye [18]. While both of these agents may downregulate the inflammation of KD, IVIG seems to demonstrate a more powerful effect on down-regulating inflammation with regard to myopia. Intraocular inflammation (vascular endothelial growth factor, interleukin 6, and matrix metalloproteinase-2) may play an important role in the development and progression of high myopia and myopic retinopathy [26]. IVIG have effective role on these inflammatory cytokine while treating with KD [27]. Based on this theory, the development of myopia may be suppressed with IVIG while treating KD.

In general, myopic prevalence increases with age in childhood as the global elongates [28]. In our study, the average age of the aspirin group was a little bit older than the IVIG group, but did not reach a clinically significant difference. Therefore, aging itself could not explain the result of higher myopic risk in the aspirin cohort. Other mechanisms, such as the anti-inflammatory property, may be considered for this outcome.

A few limitations should be considered in our study. First of all, information of these cases was retrieved from the NHIRD database. The diagnostic code used to retrieve patients’ data could not reveal the severity of KD cases and myopia, as for instance the elevation of C-reactive protein or other factors confirming KD severity as non-responsiveness to IVIG. The prolonged admission of some severe KD patients would prevent them from playing outdoors, thus reducing the protective factor for myopic occurrence [29]. Furthermore, instead of spending time in the sunlight, the load of near work, such as using a smart phone or tablet, would increase and aggravate myopic issues [3]. Second, the case numbers of these two groups demonstrate a great disparity. The diagnosis of myopia (ICD-9-CM: 367.1) is also a limitation of this study, clinical survey data of vision check were needed in the future studies.

The advantage of our study is that all the cases we recruited had a certain KD diagnosis, which could eliminate false positive results. To determine the possible mechanism behind our study result, further study of ocular images, for example, indocyanine green angiography, can be applied to evaluate any irregular perfusion or sign of choriocapillaropathy in KD cases. In conclusion, result from this analysis demonstrated that efficacy treatment with IVIG in Kawasaki disease can decrease myopic risk and having impact on myopia control. However, it needs further investigation including clinical myopia vision survey due to the limitations of this population-based study.

## Figures and Tables

**Table 1:** Demographic data of Kawasaki disease patients.

Variable	Patient with Aspirin Use		Patient with IVIG Use		*p* Value
	n	%	n	%	
Follow-up, years (means ± SD)	3.67 ± 1.34		4.00 ± 1.41		0.094
Gender					
Male	27	50.94	896	64.32	0.046 *
Female	26	49.06	497	36.68	
Age, years					
<1	14	26.42	508	36.47	0.176
1~3	26	49.06	656	47.09	
4~6	13	24.53	229	16.44	
(means ± SD)	2.00 ± 1.16		1.74 ± 1.29		0.153
Myopia					
without	36	67.92	1083	77.75	0.093
with	17	32.08	310	22.25	

* *p* < 0.05. IVIG: intravenous immunoglobulin.

**Table 2:** Comparison the incidence of myopia between aspirin and IVIG groups.

Variables	Crude HR (95% CI)	Adjusted HR (95% CI)
Kawasaki disease		
Aspirinuse	1.00	1.00
IVIG use	0.59 (0.36~0.96) *	0.62 (0.38~1.02)
Gender		
Boy	1.00	1.00
Girl	0.62 (0.49~0.79) †	0.68 (0.53~0.87) **
Age, years		
<1	1.00	1.00
1~3	1.94 (1.49~2.53) †	1.89 (1.45~2.47) †
4~6	10.94 (7.29~16.42) †	10.10 (6.71~15.19) †

* *p* < 0.05; ** *p* < 0.01; † *p* < 0.0001. HR: Hazard Ratio. IVIG: intravenous immunoglobulin.

## Data Availability

Data available by request to corresponding author.
